# Repeated Sense of Hunger Leads to the Development of Visceral Obesity and Metabolic Syndrome in a Mouse Model

**DOI:** 10.1371/journal.pone.0098276

**Published:** 2014-05-30

**Authors:** Jong-Min Han, Hyeong-Geug Kim, Jin-Seok Lee, Min-Kyung Choi, Young-Ae Kim, Chang-Gue Son

**Affiliations:** Liver and Immunology Research Center, Institute of Traditional Medicine and Bioscience of Daejeon University, 22-5, Daeheung-dong, Jung-gu, Daejeon, Republic of Korea; Hosptial Infantil Universitario Niño Jesús, CIBEROBN, Spain

## Abstract

Obesity-related disorders, especially metabolic syndrome, contribute to 2.8 million deaths each year worldwide, with significantly increasing morbidity. Eating at regular times and proper food quantity are crucial for maintaining a healthy status. However, many people in developed countries do not follow a regular eating schedule due to a busy lifestyle. Herein, we show that a repeated sense of hunger leads to a high risk of developing visceral obesity and metabolic syndrome in a mouse model (both 3-week and 6-week-old age, 10 mice in each group). The *ad libitum* (AL) group (normal eating pattern) and the food restriction (FR) group (alternate-day partially food restriction by given only 1/3 of average amount) were compared after 8-week experimental period. The total food consumption in the FR group was lower than in the AL group, however, the FR group showed a metabolic syndrome-like condition with significant fat accumulation in adipose tissues. Consequently, the repeated sense of hunger induced the typical characteristics of metabolic syndrome in an animal model; a distinct visceral obesity, hyperlipidemia, hyperglycemia and hepatic steatosis. Furthermore, we found that specifically leptin, a major metabolic hormone, played a major role in the development of these pathological disorders. Our study indicated the importance of regular eating habits besides controlling calorie intake.

## Introduction

Obesity-related disorders have become a global health problems and it was estimated 2.8 million deaths each year, with significant increasing morbidity [1]. Among these disorders, metabolic syndrome is common and includes conditions such as visceral obesity, dyslipidemia, insulin resistance and hepatic steatosis, which can result in diabetes mellitus, cardiovascular disease and stroke [2]. Consequently, there is an increasing need for studies exploring obesity control and prevention of metabolic syndrome.

Obesity occurs due to the imbalance of calorie intake and expenditure in the body. For many people, this imbalance results from excessive eating and lack of exercise. The human body is highly flexible regarding food intake and energy output within a wide calorie range. In particular, the ability to store extra calories as fat in different parts of the body has developed during starvation or lack of food intake for an extended length of time [3]. The brain regulates energy homeostasis in response to signals from both adipose tissue and the gastrointestinal tract via adjusting food intake and energy expenditure, which results in maintaining the stable body weight [4]. The several hunger hormones such as leptin, resistin and ghrelin are known to mediate these processes [5].

Frequently, people in modern society attempt to reduce their body weight using a calorie-controlled diet and intermittent fasting over a short period [6], [7]. Many clinical studies have demonstrated that uncontrolled eating habits, such as diminishing the frequency of eating, repeated fasting and recurrent overeating, were linked with obesity-related disorders [8]–[11]. For example, during the month of Ramadan, practicing Muslims abstain from eating, drinking and smoking from dawn to sunset, followed by compensatory overeating; many observers of Ramadan experience weight gain and high levels of plasma lipids [12], [13]. This pattern may be similar to the habits of people in a modern society who do not follow a regular eating schedule. Therefore, we hypothesized that a repeated sense of hunger contributed to the development of visceral obesity and metabolic syndrome.

Herein, we evaluated the repeated sense of hunger is a high-risk factor for the development of metabolic syndrome and its underlying mechanisms in animal model.

## Materials and Methods

### Animals and Experiment Design

Specific pathogen-free 3-week-old (3W; n = 20) and 6-week-old (6W; n = 20) male ICR mice were purchased from a commercial animal breeder, Daehan Biolink (Gyeongido, Korea). Mice were housed in an environmentally controlled room at 22±2°C, 55% ±10% relative humidity and a 12-h light/dark cycle. Mice were fed commercial pellets (Koatech, Gyeongido, Korea) and tap water *ad libitum* for 1 week. Total 40 mice were randomly divided into two groups consisting of 10 mice in each group: (1) the *ad libitum* (AL) food intake groups in both age of 3W and 6W (3W-AL and 6W-AL), and (2) the food restriction (FR) groups (alternate day food intake with only 1/3 of average eating amount group) in both age of 3W and 6W (3W-FR and 6W-FR) respectively. The food restriction experiment was lasted for 8 weeks. This animal experiment was approved by the Institutional Animal Care and Use Committee of Daejeon University (DJUARB2012-010) and was conducted in accordance with the Guide for the Care and Use of Laboratory Animals published by the U.S. National Institutes of Health (Bethesda, MD).

### Measurement of Food Intake, Body Weight and Organ Weight

Food intake was monitored daily and body weight was measured twice weekly. In final day, all mice were anesthetized with ether after 12 h of fasting and whole blood was collected *via* the abdominal aorta. The liver, muscle and fat pads (visceral adipose tissue, VAT; retroperitoneal adipose tissue, RAT; and epididymal adipose tissue, EAT) were removed, weighed and frozen in liquid nitrogen or stored in RNAlaterTM (Qiagen, Valencia, CA, USA). Brain was quickly removed and the hypothalamus was dissected for gene expression analysis (n = 5) or western blot analysis (n = 5). Histological examinations of samples were fixed in 10% formalin solution for 24 h.

### Serum Biomarker Analysis

All parameter concentrations were measured using sera obtained from fasting blood, which was previously clotted (15 min, room temperature) and centrifuged (15 min, 1000 × *g*). The serum samples were then frozen immediately at −80°C until further analysis. The serum levels of aspartate aminotransferase (AST), alanine aminotransferase (ALT), alkaline phosphatase (ALP), total cholesterol, high-density lipoprotein (HDL), triglycerides and glucose were determined using an autoanalyzer (Chiron, Emeryville, CA, USA).

### Determination of Serum Adipokines and Cytokines

Serum leptin, tumor necrosis factor-α (TNF-α) and interleukin-6 (IL-6) levels were measured using commercially available enzyme-linked immunoassay (ELISA) kits according to the manufacturer’s instructions (R&D Systems, Minneapolis, MN, USA). Serum ghrelin concentrations were measured using a commercial ELISA kit (RayBiotech Inc., Norcross, GA, USA) and serum levels of adiponectin and resistin were measured using a commercial ELISA kit (AdipoGen, Inc., Seoul, Korea). Standard curves were generated, from which protein concentrations were calculated.

### Determination of Lipid Level in Liver Tissue

Livers were homogenized in PBS and the protein concentrations were determined. Liver homogenate (300 µL) was extracted with 5 mL of chloroform/methanol (2∶1) and 0.5 mL of 0.1% sulfuric acid [14]. An aliquot of the organic phase was collected, dried under nitrogen and resuspended in 2% Triton X-100. Hepatic triglyceride and cholesterol content were determined using commercially available kits (Asan Pharm. Co., Seoul, Korea).

### Histopathological Analysis of Fatty Liver and Adiposity

For histopathological evaluation, freshly isolated liver was fixed in 10% formalin for 24 h. The liver was dissected under a stereomicroscope, embedded in Tissue-Tek OCT compound (Sakura Finetek, Inc., Torrance, CA, USA), and placed on a cryotome model CM 1850 (Leica Microsystems, Wetzlar, Germany). Livers were cut into 10-µm slices, mounted on slides and allowed to dry for 1–2 h. The sections were fixed in 10% formalin for 10 min and then the slides were rinsed with PBS (pH 7.4). After air-drying, the slides were placed in 100% propylene glycol for 2 min and stained in 0.5% Oil Red O solution in propylene glycol for 30 min. The slides were transferred to an 85% propylene glycol solution for 1 min, rinsed in distilled water for 2 changes and processed for hematoxylin counterstaining. For the histological evaluation, freshly white adipose tissues (VAT, RAT and EAT) were fixed in 10% formalin for 24 h. Following sufficient rinsing under flowing water, tissues were processed in a paraffin automatic processor using a programmed cascade. The paraffin-embedded samples were dissected into 4-µm-thick sections and stained with hematoxylin-eosin (H&E). After H&E staining for adipose tissues, representative histological features such as adiposity were observed under a microscope. Liver and adipose tissue images were captured with a BX51 microscope (Olympus, Japan).

### qRT-PCR Analysis

Total RNAs were extracted from liver, muscle, brain and adipose tissue samples with TRIzol reagent (Molecular Research Center, Cincinnati, OH, USA). The cDNA was synthesized from total RNA (2 µg) in a 20-µL reaction using a high-capacity cDNA reverse transcription kit (Ambion, Austin, TX, USA). Quantitative real-time polymerase chain reaction (qRT-PCR) was performed using SYBR Green PCR Master Mix (Applied Biosystems, Foster City, CA, USA) to determine gene expression for fatty acid synthase (FAS), sterol regulatory element-binding protein-1c (SREBP-1c), IL-6, proliferator-activated receptor alpha (PPAR-α), peroxisome proliferator-activated receptor gamma (PPARγ), AMP-activated protein kinase (AMPK), stearoyl-CoA desaturase-1 (SCD-1), neuropeptide Y (NPY), agouti-gene related protein (AgRP), proopiomelanocortin (POMC), melanocortin receptor-4 (MC4R), resistin, adiponectin, leptin and β-actin with the IQ5 PCR Thermal Cycler (Bio-Rad, Hercules, CA, USA). Primers used are described in Table 1. Reactions were performed with 12.5 µL of SYBRGreen PCR Master Mix, 1 µL of 10 pmol/L primer pair, 10.5 µL of distilled water and 1 µL of cDNA. Each PCR run was performed under the following conditions: initial denaturation at 95°C for 5 min and 40 amplification cycles of denaturation at 95°C for 1 min, annealing at 58°C for 40 s, and elongation at 72°C for 40 s, followed by a single fluorescence measurement. For data analysis, the gene expression levels were compared with β-actin as a reference gene.

**Table 1:** Target genes and their primary sequences.

Target genes	Accession No.	Gene sequences	
		Forward	Reverse
FAS	NM_007988	TGTGAGTGGTTCAGAGGCAT	TTCTGTAGTGCCAGCAAGCT
SREBP-1c	NM_011480	GAGCGAGCGTTGAACTGTAT	ACTTCAACGATGGGGACTTG
IL-6	NM_031168	GCTACCTGGAGTACATGAAG	CTGTGACTCCAGCTTATCTG
PPARα	NM_001113418	CCTGAACATCGAGTGTCGAA	GTACTGGCATTTGTTCCGGT
PPARγ	NM_001127330	TGGGAGATTCTCCTGTTGAC	AGGTGGAGATGCAGGTTCTA
AMPK	NM_001013367	TTGTTGGATTTCCGTAGTATTGATG	GGCAAGATCGATAGTTGCTGATG
SCD-1	NM_009127	ACGCCGACCCTCACAATTC	AGTTTTCCGCCCTTCTCTTTG
NPY	NM_023456	CGCTCTGCGACACTACATCAA	CGTTTTCTGTGCTTTCCTTCATT
AgRP	NM_001271806	GTTGCTGAGTTGTGTTCTGC	AACTTCTTCTGCTCGGTCTG
POMC	NM_001278581	ACGTGGAAGATGCCGAGATT	TTCATCTCCGTTGCCAGGAA
MC4R	NM_016977	GGTCGGAAACCATCGTCATT	TGAAATACCTGTCCACCGCA
Resistin	NM_001204959	CCACTGTGTCCCATCGATGA	GGAGGAGACTGTCCAGCAATT T
Adiponectin	NM_009605	CCTCTTAATCCTGCCCAGTCAT	GCCATCCAACCTGCACAAGT
Leptin	NM_008493	CAGAGGGTCACTGGCTTGGA	AGGCTGGTGAGGACCTGTTG
Glut4	NM_009204	CTGCTTCTGGCTCTCACAGTACTC	CCAGGTTCCGGATGATGTAGA
β-actin	NM_007393	AGGCTGTGCTGTCCCTGTATG	TGGCGTGAGGGAGAGCAT

Gene expressions were measured using visceral adipose tissues for FAS, SREBP-1c, IL-6, resistin, adiponectin and leptin, brain tissues for POMC, NPY, MC4R and AgRP, hepatic tissues for SREBP-1c, FAS, PPARγ, SCD-1, PPARα and AMPK, and muscle tissues for GLUT4, AMPK and PPARα respectively.

### Western Blot Analysis

Hypothalamus of brain tissues were homogenized in ice-cold RIPA buffer supplemented with protease (Calbiochem, San Diego, CA, USA) and phosphatase (Sigma-Aldrich, St. Louis, MO, USA) inhibitors. Protein concentrations were determined using the bicinchoninic acid (BCA) protein assay (Sigma-Aldrich). Equal amounts of protein extracts (50 µg) were fractionated by SDS-PAGE and transferred to 0.45-µm nitrocellulose membranes. Membrane blocking was performed by incubating for 1 h at room temperature with 5% nonfat dry milk in Tris-buffered saline containing 0.1% Tween-20. Then, the membranes were incubated overnight at 4°C with the POMC and actin primary antibody (Santa Cruz Biotechnology, Santa Cruz, CA, USA) at a 1∶1000 dilution. The blots were washed three times with washing buffer (20 mM Tris, 160 mM NaCl and 0.1% Tween 20), followed by a 1-h incubation with the appropriate horseradish peroxidase-conjugated secondary antibody. The peroxidase activity was detected using the Immobilon Western HRP detection reagent (Millipore, Billerica, MA, USA) using an Image Reader (Thermo Fisher Scientific, Pittsburgh, PA, USA).

### Statistical Analysis

Statistical significance was analyzed by one-way analysis of variance (ANOVA) followed by Dunnett’s post hoc test using the JMP 5.1 software (SAS Institute, Cary, NC, USA). The results are expressed as means ± standard deviation (SD). In all analyses, *p<0.05 or **p<0.01 indicated significance.

## Results

### Food Intake and Weight of Body, Liver and Adipose Tissues

**Figure 1:**
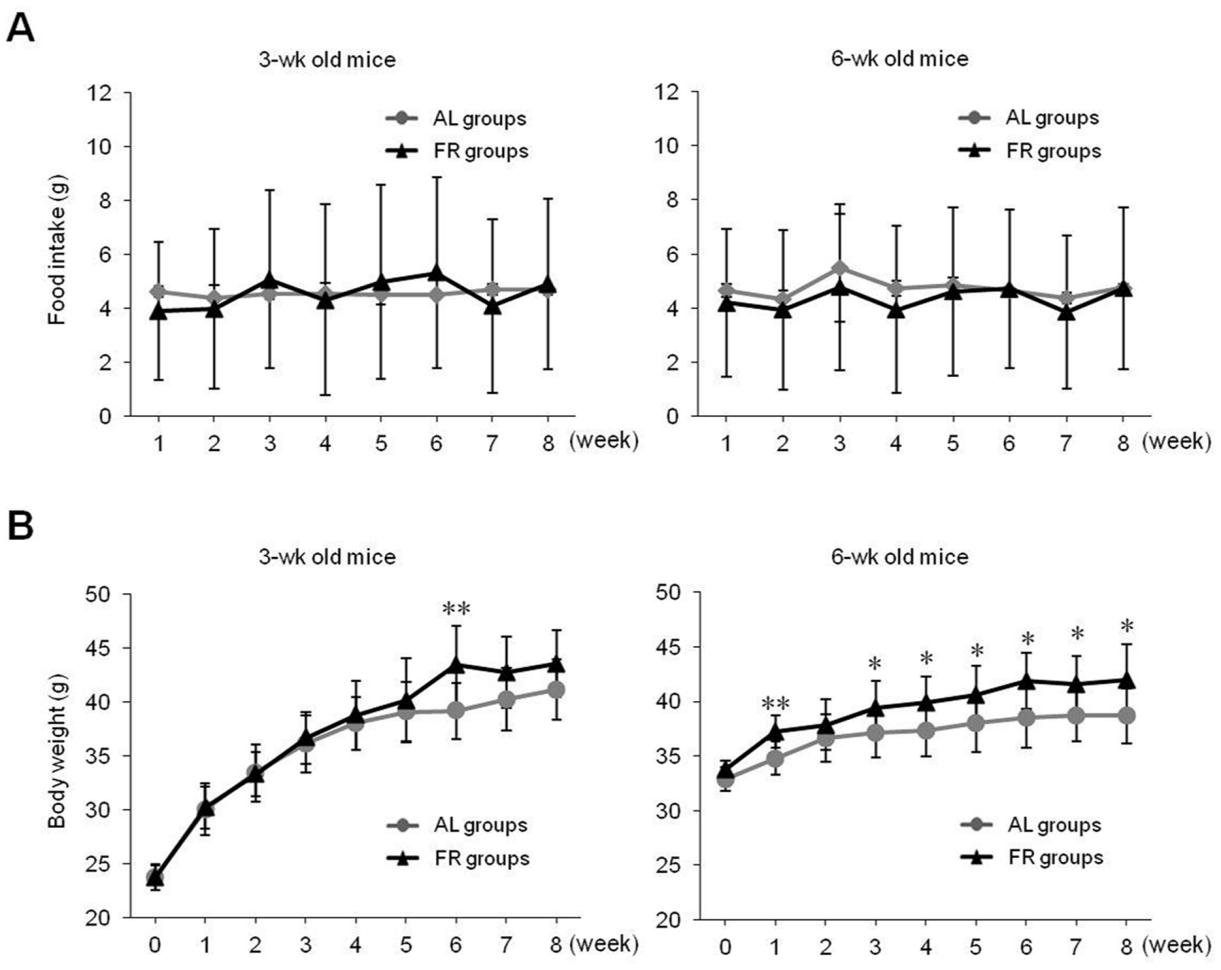
Food intake and body weight. (A) Food intake was monitored daily. (B) The body weight was measured twice weekly. Each point represents the mean ± standard deviation (SD; n = 10). *p<0.05, **p<0.01 compared with the AL group.

**Figure 2:**
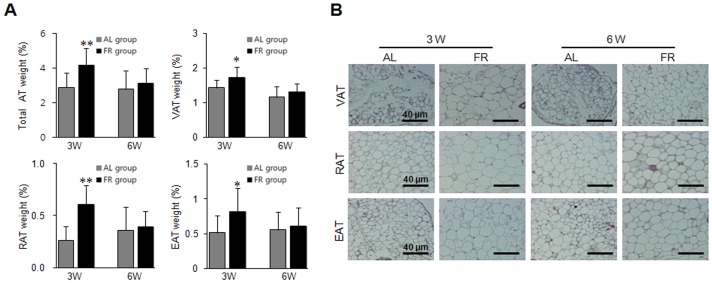
Fat pad weight and histological findings of adipose tissues. (A) The fat pads were weighed from visceral adipose tissue (VAT), retroperitoneal adipose tissue (RAT) and epididymal adipose tissue (EAT). Data are expressed as means ± SD (n = 10). *p<0.05, **p<0.01 compared with the AL group. (B) After hematoxylin and eosin (H&E) staining, the histological differences of adipose tissues were examined under a microscope (Scale bars: 40 µm).

After the 8-week experiment using two different feeding conditions, the food intake per week and throughout the 8-week experiment in the FR groups was nearly identical or slightly lowers than AL groups in 3W and 6W mice, respectively (Figure 1A and Table 2). However, the body weight and weight gain were higher in the FR groups compared to the AL groups on final day (*p*<0.05 in 6W group, Figure 1B and Table 2). Additionally, the relative liver weight was significantly higher in the FR groups compared with the AL groups (*p*<0.01, Table 2). The total weight of adipose tissues was significantly higher in the FR group than the AL group, particularly in 3W mice (*p*<0.01, Figure 2A). The same patterns were observed for each regional analysis of VAT (*p*<0.05 in 3W group), RAT (*p*<0.01 in 3W group), and EAT (*p*<0.05 in 3W group, Figure 2A). Histological findings revealed the size of adipocytes was markedly increased in the FR groups compared with the AL groups (Figure 2B).

**Table 2:** Food intake, body and liver weight, and serum biochemistry parameters.

Groups		3W		6W	
		AL (n = 10)	FR (n = 10)	AL (n = 10)	FR (n = 10)
Food intake (g/day)		4.56±0.25	4.56±3.23	4.73±0.43	4.34±2.95
Body weight	Initial day (g)	23.7±1.2	23.7±1.1	32.9±1.1	33.1±0.7
	Final day (g)	41.2±2.8	43.5±3.1	38.7±2.5	42.1±2.85*
	Weight gain (g)	17.4±3.3	19.8±3.5	5.8±16.7	8.7±3.0*
Liver weight	Absolute (g)	1.5±0.1	1.7±0.1**	1.33±0.14	1.63±0.14**
	Relative (%)	3.65±0.29	3.99±0.13**	3.44±0.26	3.90±0.46*
Total cholesterol (mg/dL)		112.6±14.6	131.2±13.7*	122.7±10.1	140.1±17.0*
LDL (mg/dL)		26.6±10.8	30.0±5.9	30.5±6.6	44.2±10.7*
HDL (mg/dL)		73.7±8.2	79.5±6.7	73.2±16.7	78.9±12.1
Triglyceride (mg/dL)		93.7±17.2	91.2±9.1	96.2±12.4	85.0±5.3
Glucose (mg/dL)		79.2±12.35	88.3±7.9	78.5±12.8	95.0±9.6*
AST (IU/L)		58.0±10.8	58.3±16.3	48.5±12.4	66.8±16.2*
ALT (IU/L)		29.5±10.6	39.9±13.7	26.2±11.5	38.7±24.7
ALP (IU/L)		43.8±10.5	55.0±16.2	33.1±14.1	36.8±9.9

Data are expressed as means ± SD. *p<0.05, **p<0.01 compared with the AL group.

### Serum Levels of Metabolic and Inflammatory Mediators

**Figure 3:**
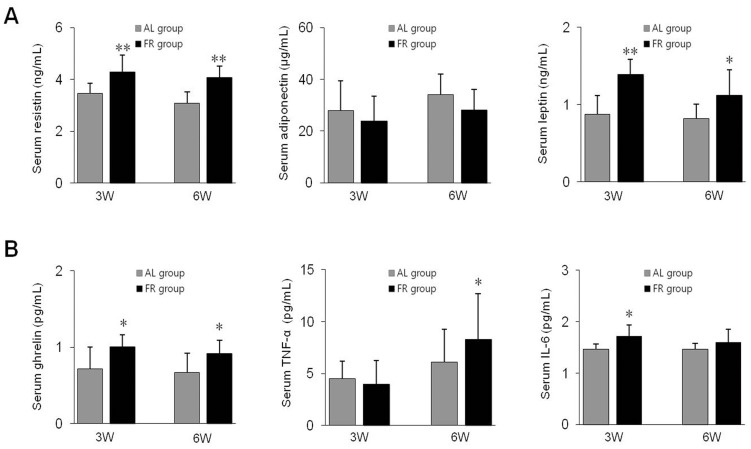
Serum protein levels of metabolic and proinflammatory mediators. (A) The protein levels of resistin, adiponectin and leptin as well as (B) ghrelin, tumor necrosis factor-α (TNF-α) and interleukin-6 (IL-6) in serum were analyzed by ELISA. Data are expressed as means ± SD (n = 10). *p<0.05, **p<0.01 compared with the AL group.

The serum resistin levels were significantly higher in the FR groups compared to the AL groups (*p*<0.01). Serum adiponectin levels were slightly lower in the FR groups (*p*>0.05), whereas leptin levels were significantly increased in the FR groups compared to the AL groups (*p*<0.05 in 6W group, *p*<0.01 in 3W group, Figure 3A). The serum ghrelin levels were significantly increased in the FR groups compared to the AL groups (*p*<0.05, Figure 3B).

In addition, the serum levels of the inflammatory cytokines, TNF-α and IL-6, were significantly higher in the FR groups compared with the AL groups (*p*<0.05 in 6W group for TNF-α, and *p*<0.05 in 3W group for IL-6, Figure 3B).

### Serum Levels of Lipid Profiles and Glucose

The FR groups showed high levels of total cholesterol (*p*<0.05) and low-density lipoprotein (LDL) cholesterol (*p*<0.05 in 6W group, Table 2). However, no significant difference was observed in HDL and triglycerides between the two groups. The FR groups showed a pattern of high glucose level compared with the AL groups (*p*<0.05 in 6W group, Table 2).

### Adipogenic and Metabolic Gene Expressions in Adipose Tissues

**Figure 4:**
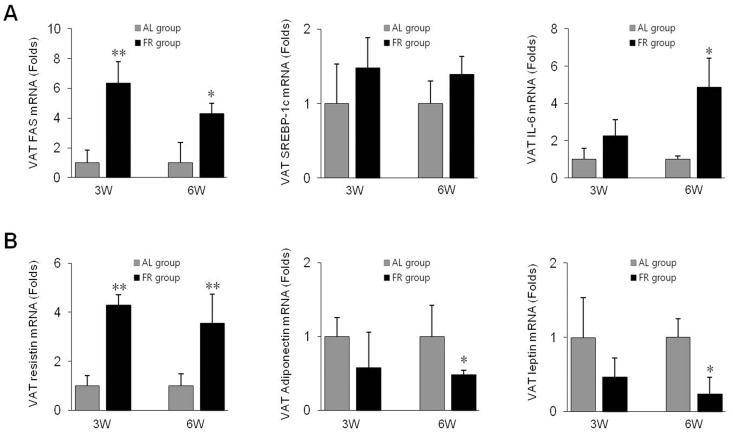
Gene expression levels in visceral adipose tissue (VAT). (A) The mRNA expression levels of fatty acid synthase (FAS), sterol regulatory element-binding protein-1c (SREBP-1c) and interleukin-6 (IL-6) as well as (B) resistin, adiponectin and leptin were determined using qRT-PCR analysis. Data are expressed as means ± SD (n = 10, fold change relative to the AL group). *p<0.05, **p<0.01 compared with the AL group.

Based on the mRNA levels of fat accumulation-related and adipokine genes in VATs, the expression levels of FAS and SREBP-1c were significantly upregulated in the FR groups compared to the AL groups (Figure 4A). In addition, the mRNA level of resistin was significantly upregulated (*p*<0.01), whereas those of adiponectin and leptin were downregulated in the FR groups compared to the AL groups (*p*<0.05 in 6W group, Figure 4B).

### Glucose Uptake-related Gene Expressions in Muscle Tissues

**Figure 5:**
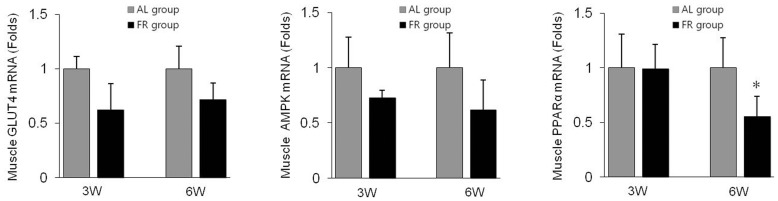
Gene expression levels in skeletal muscles. The mRNA levels of glucose transporter 4 (GLUT4), AMP-activated protein kinase (AMPK) and proliferator-activated receptor alpha (PPARα) were determined using qRT-PCR analysis. Data are expressed as means ± SD (n = 10, fold change relative to AL group). *p<0.05 compared with the AL group.

Based on the expression levels of glucose uptake genes in femoral muscle tissues, FR groups showed downregulated expression of glucose transporter 4 (GLUT4), AMPK and peroxisome PPARα compared with the AL groups. However, only PPARα expression showed a significant difference between the two groups (*p*<0.05 in 6W group, Figure 5).

### Lipid Levels, Inflammation Enzymes and Lipogenic Gene Expression in Hepatic Tissues

**Figure 6:**
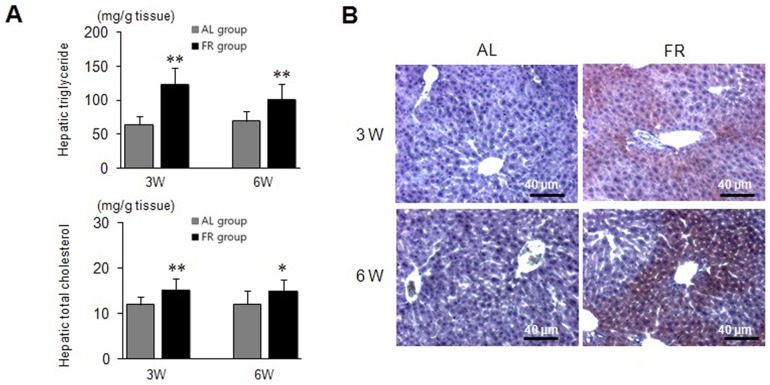
Measurement of hepatic steatosis. (A) Hepatic triglycerides and cholesterol were measured using commercially available kits. Data are expressed as means ± SD (n = 10, respectively). *p<0.05, **p<0.01 compared with the AL group. (B) Hepatic tissues were evaluated using Oil Red O staining and the histological differences were examined under a microscope (Scale bars: 40 µm).

The FR groups showed significant hepatic steatosis, as evidenced by the increased hepatic triglycerides (*p*<0.01) and cholesterol (*p*<0.05 in 6W group, *p*<0.01 in 3W group) levels compared to the AL groups (Figure 6A); moreover, larger lipid droplets were observed in the histopathological findings (Figure 6B). The FR groups showed elevated hepatic enzyme levels (AST, ALT and ALP) compared to the AL groups (*p*<0.05 in 6W group for AST, Table 2).

**Figure 7:**
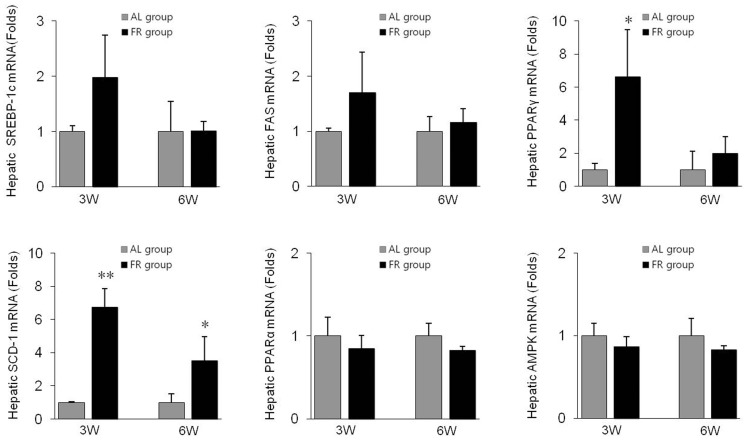
Gene expression levels in the liver. The mRNA levels of sterol regulatory element-binding protein-1c (SREBP-1c), fatty acid synthase (FAS), proliferator-activated receptor gamma (PPARγ), stearoyl-CoA desaturase-1 (SCD-1), proliferator-activated receptor alpha (PPARα) and AMP-activated protein kinase (AMPK) were determined using qRT-PCR. Data are expressed as means ± SD (n = 10, fold change relative to the AL group). *p<0.05, **p<0.01 compared with the AL group.

In addition, the FR groups showed marked upregulation of hepatic lipogenic genes, including SREBP-1c, FAS, PPARγ and SCD-1, compared to the AL groups, while the energy expenditure-related genes, PPARα and AMPK, were downregulated in the FR groups (Figure 7).

### Protein and Gene Expression Levels of Appetite-related Neuropeptides in Brain Tissues

**Figure 8:**
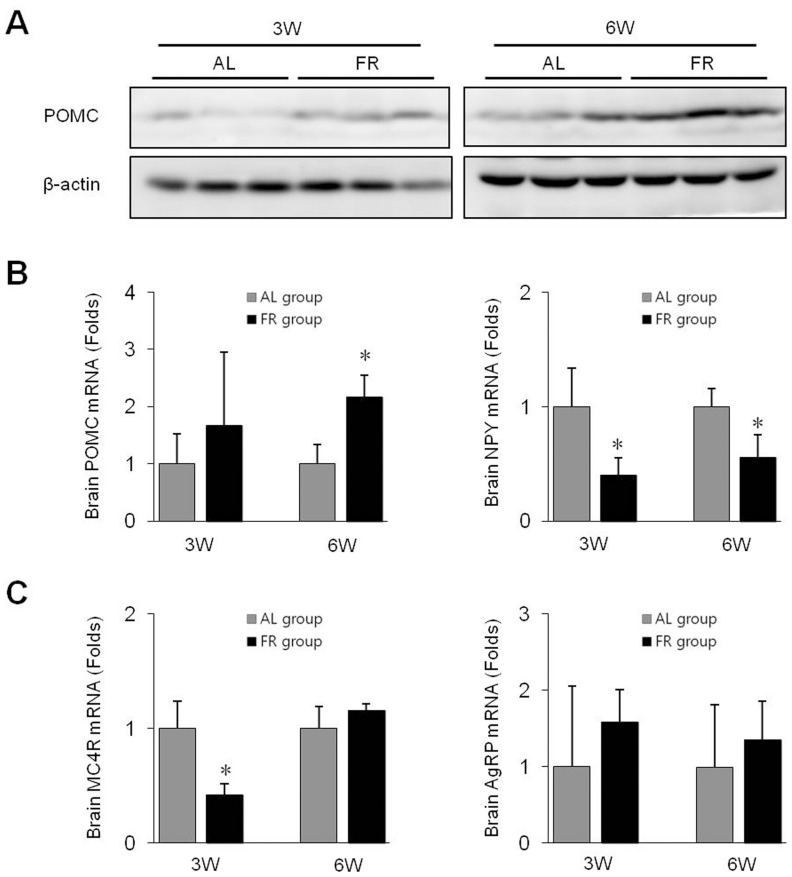
Protein and gene expression levels in the brain. (**A**) Proopiomelanocortin (POMC) activity was analyzed by Western blotting (n = 5). (**B**) The mRNA levels of POMC and neuropeptide Y (NPY) as well as (**C**) melanocortin receptor-4 (MC4R) and agouti-gene related protein (AgRP) were determined using qRT-PCR. Data are expressed as means ± SD (n = 5, fold change relative to the AL group). *p<0.05 compared with the AL group.

The expression of the anorexigenic POMC was highly activated at both the protein and mRNA levels in the FR groups compared with the AL groups (Figure 8A and B), and the expression of the orexigenic NPY gene was downregulated in the FR groups. Regarding the two other hunger-related genes, the expression of MC4R was significantly downregulated (*p*<0.05 in 3W group), while that of AgRP was not changed significantly in the FR group (Figure 8C).

## Discussion

A few clinical data anticipated that eating at regular times and proper food quantity are crucial for maintaining a healthy status [8], [9]. However, many people in developed countries do not follow a regular eating schedule due to a busy lifestyle. Herein, we showed in a mouse model that a repeated sense of hunger led to a high risk for the development of visceral obesity and metabolic syndrome. At the end of 8 weeks, the total food intake in the FR groups (subjected to an 8-week repeated pattern of partial restriction diet on one day followed by compensatory overeating the next day) was slightly lower than the AL groups (fed daily using a normal feeding pattern). However, the final body weight and weight gain were higher in the FR groups than the AL groups. Consequently, the FR groups showed pathological conditions characteristic of metabolic syndrome, including a distinct visceral obesity, hyperlipidemia, hyperglycemia and hepatic steatosis.

Metabolic syndrome is a disorder that includes several conditions; e.g., obesity, dyslipidemia and hyperglycemia [15]. Obesity is a principle causative factor in the development of metabolic syndrome and is related to a higher level of lipid accumulation in liver, skeletal muscle and adipose tissues [16], [17]. The FR groups showed significantly elevated serum levels of total cholesterol and LDL cholesterol in our study. In obese conditions, adipocytes accumulate large amounts of lipids and become enlarged. Especially in obese adults, the change in adipocyte size is more important than the number of adipocytes [18]. As expected, VAT weight was significantly increased in the FR groups, which was in accordance with the histological analysis, showing a markedly enlarged size of adipocytes. Lipogenesis generally occurs in differentiated adipocytes, which store fatty acids in the form of triglycerides *via* several cytoplasmic enzymes including SCD-1, SREBP-1c, and FAS [19]. In our study, the mRNA expression levels of FAS and SREBP-1c in adipose tissues were significantly upregulated in the FR groups compared to the AL groups.

Three main adipokines, resistin, adiponectin and leptin are adipocyte-secreted molecules that act as core contributors to the development or regulation of obesity, insulin resistance and hepatic steatosis [20]. Resistin plays a central role in the differentiation of adipocytes leading to increased obesity and insulin resistance [21], [22], while adiponectin acts as a potent inhibitor of the inflammatory reaction in obese subjects [23]. A high serum resistin level but low adiponectin level has been noted in an obese population [24]. Leptin plays a key role in the long-term regulation of body weight and energy homeostasis *via* control of appetite [25], [26]. As our expectation, results from our study showed significantly high serum resistin levels but low adiponectin levels in the FR groups compared to the AL groups. Additionally, serum leptin levels were significantly increased in the FR groups. A previous report demonstrated that serum leptin levels were significantly higher in an obese group than a lean group, with a close correlation to body weight [27]. Thus, leptin is a significant indicator of metabolic syndrome, and the same relationship between leptin and obesity creates a high risk factor for the development of metabolic syndrome [28]. Ghrelin is known as a hormone maintaining appetite leading to storage of energy accumulation in adipose tissue, similar to adipokines [29], [30]; our results showed higher serum ghrelin levels in the FR groups.

Previous studies demonstrated that fat accumulation leads to chronic inflammation during obesity progression [31], [32]. The representative inflammatory cytokines, TNF-α and IL-6, were increased under the obesity condition. Along with adipokines, these two cytokines are recognized as markers of the metabolic syndrome-like condition, characterized by abdominal obesity, hyperlipidemia and hyperglycemia [33], [34]. In our study, the FR groups showed significantly higher levels of serum TNF-α and IL-6 compared to the AL groups.

Excess visceral fat also increases the risk for the development of insulin resistance, a typical feature of metabolic syndrome [35], [36]. Moreover, an elevated serum leptin level is known to correlate closely with insulin resistance [37]. In our results, serum leptin and glucose levels in the FR groups were significantly elevated compared to the AL groups. A human clinical trial showed that the relationship between leptin and glucose serum levels was more significant in an older population [38], similar to our results between the 6W and 3W groups. GLUT4 is a glucose transport protein found in adipose tissues and skeletal muscle [39]. The gene expression level of GLUT4 in skeletal muscle was reduced under the diabetic condition in an animal model [40] as well as in humans [41]. GLUT4 is regulated by AMPK and PPARα, and considerable evidence supports the AMPK-mediated process protects against insulin resistance [42]. We investigated the GLUT4 expression in femoral muscle tissue, which provided additional information regarding the high glucose level. The FR groups showed a marked downregulation of GLUT4 mRNA levels in muscle compared with the AL groups. Similar results were observed in AMPK and PPARα levels in the FR groups compared with the AL groups.

The liver is a central organ of fat metabolism and hepatic steatosis and consequential liver injury are observed frequently in patients with metabolic syndrome [43]. High serum leptin levels and insulin resistance are contributors to the pathogenesis of hepatic steatosis [44]. The FR groups showed significant hepatic steatosis evidenced by the notable lipid droplets and the increase of hepatic triglycerides and cholesterol levels compared to the AL groups. The major lipogenic genes, SREBP-1c, FAS, SCD-1 and PPARγ are involved in *de novo* lipogenesis in hepatic tissues [45]. Noticeable upregulation of hepatic lipogenic genes including SREBP-1c, FAS and SCD-1 was evident in the FR groups, while the energy expenditure-related genes, PPARα and AMPK were downregulated.

**Figure 9:**
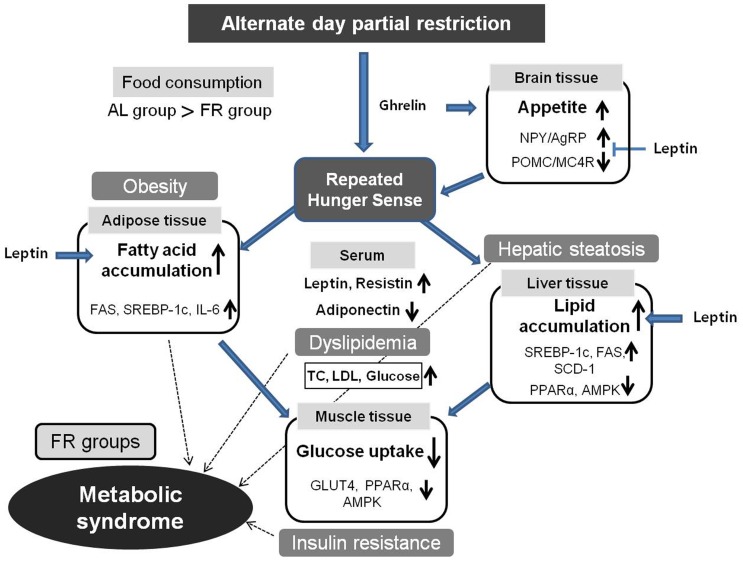
Graphical summary of the metabolic syndrome by a repeated hunger sense.

The above results suggest that a repeated sense of hunger alters the quantitative changes in adipokines as well as cytokines leading to metabolic-syndrome-like conditions such as abdominal obesity, dyslipidemia, hepatic steatosis and hyperglycemia tendency (Figure 9). This pathological state is thought to be linked to diet control in the brain. The brain responds to hunger *via* several neuropeptides/receptor interactions that originate from the hypothalamus such as the orexigenic NPY-AgRP neurons and the anorexigenic POMC-MC4R neurons [46], [47]. The sense of hunger increases the NPY activity but decreases the POMC activity, which leads to increased appetite [48], [49]. The partial food restriction on one day was followed by compensatory overeating on the next day; accordingly we expected the sense of hunger to be activated. However, the FR groups showed highly activated POMC but suppressed NPY, which resulted from the elevated serum leptin levels. The chronic elevation of serum leptin concentration inhibits NPY production and increases POMC in the hypothalamic neurons [49], [50].

These findings are based on an animal model, which has inevitable limitations; however our data were obtained from duplicated experiments (using 3- and 6-week-old mice) as well as another BALB/c mice model. Recently, modified diet patterns have been considered for reducing calories, including skipping regular meals, alternate day fasting or intermittent fasting. These are effective in reducing body weight and increasing longevity [51], [52]. However, uncontrolled eating habits with alternate day compensatory overeating could evoke metabolic syndrome-like disorders. Accordingly, we can conclude that the repeated sense of hunger in an animal model induced the typical characteristics of metabolic syndrome; distinct visceral obesity, hyperlipidemia, hyperglycemia and hepatic steatosis and the main underlying mechanisms involve the imbalance of adipokines, particularly leptin. Our findings are first experimental evidence to clearly show the importance of maintaining a regular daily meal schedule for the prevention of metabolic syndrome.
